# Transforming the Management of Articular Fractures in the Foot: A Critical Examination of Current Methods and Future Directions: A Review

**DOI:** 10.3390/jpm14050525

**Published:** 2024-05-15

**Authors:** Robert Daniel Dobrotă, Adrian Gheorghe Barbilian, Corina Sporea, Dumitru Ferechide

**Affiliations:** 1 Faculty of Medicine, University of Medicine and Pharmacy “Carol Davila”, 37 Dionisie Lupu Street, 020021 Bucharest, Romania; robert-daniel.dobrota@drd.umfcd.ro (R.D.D.); adrian.barbilian@umfcd.ro (A.G.B.); dumitru.ferechide@umfcd.ro (D.F.); 2 Faculty of Midwifery and Nursing, University of Medicine and Pharmacy “Carol Davila”, 37 Dionisie Lupu Street, 020021 Bucharest, Romania; 3 National University Center for Children Neurorehabilitation “Dr. Nicolae Robanescu”, 44 Dumitru Minca Street, 041408 Bucharest, Romania

**Keywords:** fracture management, foot fractures, minimally invasive, bone healing, rehabilitation protocols, personalized treatment, new technologies

## Abstract

This study provides a comprehensive examination of the current methodologies and potential strategies for the treatment of articular fractures of the foot. In the field of orthopedic healthcare, these fractures present a significant challenge due to their complex nature and the fact that they affect the routines of patients. The motivation behind this study is based on two main concepts. The first one is represented by the use of emerging medical technologies and personalized medicine to bring a significant transformation in the management of foot fractures and give a better quality of treatment that is accepted by the patient. However, because there are inequities in the availability of the necessary medical care and equipment, as well as uneven incorporation in clinical settings, new technologies cannot be used to treat these types of fractures. Regarding the second concept behind this study, it is indicated that although current treatment methods are essential, they have a number of shortcomings when it comes to properly addressing these types of injuries. An approach is needed that takes into account the biomechanical points of view and the particularities of each patient. This approach could be applied in all hospital settings. Through this study, we want to highlight the progress made in recent years in surgical techniques such as 3D printing, minimally invasive surgery (MIS), and biological products. However, in the application of this new discovery, new obstacles have been discovered that prevent the efficient treatment of these types of injuries. This study examines the effectiveness and limitations of current treatments, as well as how differences in healthcare, such as available equipment, training of medical staff, and technological advances, affect patient outcomes in everyday life. This research wishes to emphasize that continuous innovation, interdisciplinary collaboration, and the use of an optimal approach that is appropriate for each patient, are essential. This study aims to provide new insights and useful recommendations for future research and clinical practice. The main role of this research is to improve the quality of life of patients and increase the standards of care in this complex field, which is in permanent evolution.

## 1. Introduction

Given the significant impact of foot fractures on patients’ mobility and overall well-being, it is crucial to improve surgical interventions for these injuries. Adequate treatment of these fractures is crucial for guaranteeing lasting results for patients, not just faster recovery, as this can cause adverse effects in the near or distant future. That is why surgical interventions, either minimally invasive or more extensive, should be considered. The study by Chapman et al. [1] pointed out that the impact of surgical interventions, such as arthroscopically-assisted cartilage implantation, in terms of their overall effectiveness and patient satisfaction, is much improved after they are performed. However, according to Kim et al. [2], insufficient or inappropriate treatment following total ankle arthroplasty can lead to various complications, such as lack of union, malunion, persistent pain, and ultimately, arthrosis. Over time, these complications will lead to a high degree of fracture appearance and have a negative impact on general quality of life for the patient, as well as necessitating more attention from medical staff, because they will require additional long-term treatments to mitigate the side effects. Therefore, it is necessary to improve our approaches to the management of foot fractures to promote the long-term well-being of patients and reduce the number of patients with side effects presenting to various health care services.

The strategies used by each individual health system, together with the integration of technology and innovation, significantly influence clinical outcomes in the field of orthopedic treatment, especially in the field of foot fracture therapy. Bronwyn Spira [3] emphasizes the importance of recognizing and addressing health disparities as a crucial aspect of these strategies. To eliminate disparities in health care delivery, health systems must adopt an approach that identifies barriers to equitable access. These disparities are evident in the varying success rates of surgical interventions and treatment outcomes over an extended period of time, which are influenced by factors such as race, socioeconomic status, education, and other demographic variables. It is very important to consider good management of foot fractures, because access to appropriate treatment is necessary to lower the risk of future disability. Digital solutions to various health problems have become increasingly common due to the COVID-19 pandemic. Research conducted by Hofmann et al. [4] demonstrated the fact that the consultations offered in the online environment, as well as the treatments provided by the medical staff, stimulated the relationship between the patient and the doctor and optimized the use of healthcare resources, especially in the healthcare systems that are already overpopulated by patients. In addition, it has been observed that the integration of artificial intelligence (AI) into orthopedics has led to significant advances in sharing patient information, providing clinical decision support, and optimizing treatment. Giorgino et al. [5] studied the use of applications such as ChatGPT, suggesting they will improve future success in the correct diagnosis of various pathologies, but will also be able to produce appropriate and personalized treatments for each individual. The implementation of solutions based on artificial intelligence requires a cautious and conscientious approach, due to the presence of problems related to data quality, confidentiality, and ethics, but also due to the fact that most of the solutions presented by AI models are developed by various people who published them in the online environment. Thus, in the future, we want an optimal implementation of artificial intelligence into orthopedics, but with the solutions to current problems. The goal of this approach is to make these innovations more accessible and beneficial to all patients, improving foot fracture outcomes. Significant progress has been made in the treatment of articular fractures of the foot over time. Traditionally, the main method of treatment for these fractures was immobilization with plaster casts. Their purpose is to provide proper alignment and stability, so that the affected bone or bones can heal on their own. As surgical techniques have advanced, these treatments have been improved over time. More precise and less invasive treatments are now possible for the treatment of leg fractures, such as minimally invasive surgery, arthroscopy, and improved fixation devices. Notable developments, such as the development of materials used to create implants that are compatible with the body, and the use of growth factors and stem cell therapy, have also had a notable effect on treatment methods. As a result of these advances, new possibilities have emerged for better healing and rehabilitation. To improve patient outcomes, practitioners are generally open to new ideas and methods, bringing new research and practice into their work. Studies that have had a significant impact on current practice include those that have examined the biomechanics of the foot and the outcomes of various surgical techniques. Better, more targeted treatment methods are now possible because of the information gained from these studies. Improving patient care has always driven the history of continuous learning and adaptation in the field of orthopedics, especially in foot fractures.

Current treatments for leg fractures have a number of recognized limitations that can significantly affect patient outcomes. But, in addition to the treatments that require continuous development, the medical recovery of the affected segment is as important as the chosen method, if not even more so. The research carried out by Bolovan et al. [6], which investigated the effectiveness of physical training programs using customized foot orthoses versus physical training in which no such orthoses are used, represents a study that medical personnel should focus on to improve long-term outcomes, and not only short-term outcomes at the time of the trauma. Although, for a complete optimization of the appropriate treatment, the possible biological complications at the local level, or of the whole organism, must also be taken into account. The possibility of venous thromboembolism after foot or ankle fractures is another important aspect, as highlighted in the research of Gouzoulis et al. [7]. The results of this study highlight the fact that, although there is a low rate of venous thromboembolism in ankle and foot fractures, it should not be neglected, because it can even be produced by the surgical interventions that are used in the treatment of the respective pathology. Thus, it must be taken into account that the surgical interventions can have increased efficiency in the treatment of the affected bone, but can cause complications that can produce new pathologies for the patient. Shauly et al. [8] demonstrated that foot fractures with severe traumatic injuries which require reconstruction of the lower limb have a low success rate compared to other traumatic injuries, with 30% of patients needing mobility assistance 6 months postoperatively. These insights highlight the need for continued improvement and innovation in the field of foot fracture treatment to improve patient outcomes.

The main objective of this review article is to provide a thorough analysis of the current methods of treating articular fractures of the foot, as well as to suggest directions for further research and their use in the clinic. This paper has six important objectives which will be discussed in the following paragraphs, namely: diagnostic approaches, treatment modalities, rehabilitation strategies, new materials and technologies, patient outcomes, and research gaps. The general purpose of this study is to show how important it is to constantly change and improve treatment methods in light of the development of new technologies and evolving dynamics of healthcare. By providing a comprehensive and up-to-date synthesis of current practices and future perspectives, this review hopes to add significantly useful information to existing research. This article examines these views in an effort to produce more research and discussion, with the long-term goal of improving the treatment of patients with foot fractures and, more generally, potentially reshaping how trauma is viewed in orthopedics.

## 2. Research Questions and Hypothesis

The main approach of this article is divided into four key questions:

1. What are the current best practices in the management of foot fractures and where are the deficiencies? This question aims to critically analyze existing treatment modalities, identifying their strengths and weaknesses in the context of patient outcomes;
2. How can emerging technologies and innovations in medical science be integrated into the treatment of foot fractures to improve clinical outcomes? This question explores the potential of new technological advances such as artificial intelligence, telemedicine, and advanced surgical techniques;
3. What role do health disparities play in the treatment of foot fractures and how can these be addressed to ensure equal care for all patients? This question aims to understand the impact of socioeconomic and geographic factors on the treatment and outcomes of foot fractures, proposing strategies to eliminate disparities;
4. How do post-treatment medical recovery strategies and patient compliance affect long-term outcomes of foot fracture management? This question focuses on the post-treatment phase, exploring the role of rehabilitation and patient adherence in the recovery process. It aims to assess the effectiveness of current medical recovery protocols, the challenges in ensuring patients adhere to these protocols, and the long-term impact of these factors on recovery quality.

To guide this research, the article posits two hypotheses:

**Hypothesis** **1:** *The first hypothesis states that developments in medical technology, personalized medicine, and materials science are likely to lead to dramatic changes in the treatment outcomes of foot fractures. However, institutional health care problems, inconsistent integration into clinical practice, and a lack of thorough and standardized treatment regimens currently limit the full potential of these advances. If these predictions on the treatment of leg fractures are to be put into practice, we must remove these obstacles;*

**Hypothesis** **2:** *Despite their fundamental nature, current methods of treating leg fractures are inadequate to deal with the complexity of these injuries. There is an urgent need for new approaches in both surgical and non-surgical treatments, based on evolving understanding of biomechanics and patient comorbidities, with regard to compliance and post-treatment rehabilitation. This theory proposes that people with leg fractures will have better long-term outcomes and better management if their therapy is multidimensional and factors in these new findings.*

## 3. Literature Review Methodology

### 3.1. Overview and Objectives

The literature review aims to go carefully through the field of orthopedic research, specifically targeting the management of articular fractures in the foot, because the main problems for these patients are related to their routine, including walking, running, or doing any type of exercise without feeling any leg pain. This research is guided by several detailed objectives, structured to encompass existing knowledge and to determine the creation of new research initiatives.

#### 3.1.1. In-Depth Analysis of Diagnostic Approaches

In order to evaluate the evolution and current status of diagnostic techniques for foot fractures, a review of the current methods used for diagnosis is necessary. This includes examining advances in imaging technologies, from conventional radiography to cutting-edge MRI and CT, and their impact on the accuracy of fracture identification and classification. Also, due to poverty in different countries, it must be taken into account that in most emergency rooms, the only way to diagnose a pathology is to use a medical examination or X-rays, without the possibility of using advanced technologies such as CT or MRI.

#### 3.1.2. Surgical and Non-Surgical Treatment Modalities

It is necessary to analyze the treatment methodologies in detail, examining the results, complications, and recovery times. This involves a comparative analysis of traditional open reduction and internal fixation (ORIF) techniques versus minimally invasive surgical methods, as well as evaluating the role of conservative treatments such as immobilization and physical therapy.

#### 3.1.3. Rehabilitation Strategies Post-Treatment

In order to explore post-surgical rehabilitation, identifying protocols that optimize recovery and restoration of function is necessary. Special attention will be paid to the integration of physical therapy, orthotic support, and patient education in improving the quality of life after injury.

#### 3.1.4. Exploration of New Materials and Technologies

The contributions of material sciences and technological innovations in orthopedic surgery must be reviewed. This includes the use of biocompatible implants, the application of 3D printing for personalized surgical planning, the creation of implants, and the exploration of biologics and tissue engineering for improved fracture healing.

#### 3.1.5. Evaluation of Patient Outcomes and Quality of Life

To analyze patient-reported outcome data, focusing on the effectiveness of different management strategies in restoring foot function, reducing pain, and improving overall quality of life, this objective aims to correlate specific treatment modalities with long-term outcomes, taking into account both physical and psychological impacts.

#### 3.1.6. Identification of Research Gaps and Future Directions

To identify the gaps in current research and practice, proposing areas for future investigation, this objective includes assessment of unmet clinical needs, underexplored treatment methods, and the potential for new technologies to address existing challenges in the management of foot articular fractures.

By setting these objectives, the literature review aims to highlight the current state of foot fracture management, but also anticipates the trajectory of future research and clinical practice. This detailed research is intended to contribute significantly to knowledge, guiding clinicians, researchers, and decision-makers in optimizing the care of patients with these complex injuries.

### 3.2. Data Sources and Search Strategy

This literature review is based on a meticulous selection of databases, known for their comprehensive coverage of medical and scientific research. These include PubMed, Embase, Scopus, Web of Science, and Google Scholar.

In the selection of the articles, all authors were involved. To avoid divergences, the following strategy was applied:

1. Clear criteria and objectives were established, such as relevance for the topic addressed, actuality, methodological quality, and impact in the field;
2. After that, two of the authors used the same previously established search strategy, with mutual agreement, to find the most suitable articles;
3. The selected articles were reviewed by the other two authors, to validate and consolidate the selection process.

The search strategy is designed to be both complete and precise, using a combination of keywords and phrases, along with Boolean operators, to filter search results effectively. The key steps are:

Keyword development: Initial keywords are based on the main topics of interest-“fractures”, “foot”, and “management”. These are expanded by adding related terms and synonyms to find the widest possible range of relevant literature. Examples include “tarsal fractures”, “metatarsal fractures”, “ankle fractures”, “orthopedic surgical techniques”, “non-surgical treatment”, “rehabilitation”, and “innovations in fracture management”. Thus, 6898 scientific articles resulted after these criteria had been used (PubMed: 2286, Embase: 551, Scopus: 182, Web of Science: 479, Google Scholar: 3400).

Reference tracking: Review bibliographies of selected articles for additional studies that may not have been identified by database searches. Thus, 91 articles were added, totaling 6989 papers.

Duplicate removal: The duplicates were removed, obtaining 5734 scientific articles.

Boolean operators: AND was used to combine different concepts (e.g., “leg” AND “joint fractures”), and OR to include synonyms or related terms (e.g., “surgical management” OR “non-surgical management”). NOT can be used sparingly to exclude irrelevant topics. After adding this operator, we obtained a total of 4671 scientific articles. 

Search filters: Filters were applied to determine better search results, which may include language (English), publication date (last 25 years to ensure timeliness, 1999–2024), and document type (peer-reviewed articles, clinical trials, meta-analyzes, and review articles). The filters lowered the number to 239 papers.

Selection: Titles and abstracts of retrieved articles were screened for relevance based on predefined inclusion and exclusion criteria. The full texts of potentially relevant articles were then reviewed for final inclusion in the literature review. Thereby, 154 articles were removed, with only 45 eligible papers remaining.

**Figure 1:**
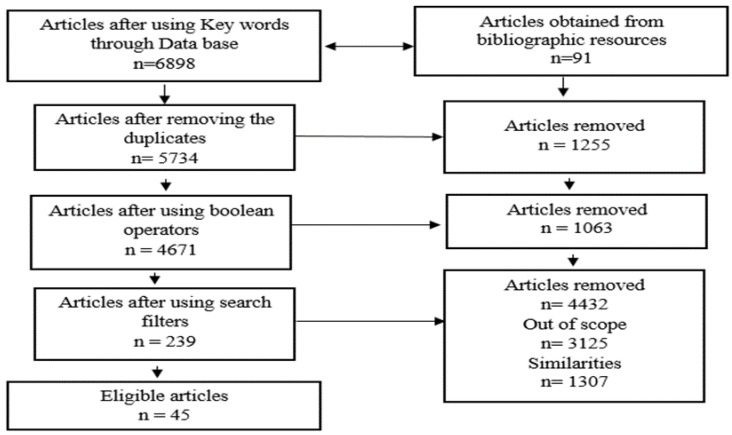
Workflow diagram.

The entire process of selection is presented in Figure 1.

This detailed approach to data sources and search strategy provides a comprehensive and systematic review of the literature on foot fracture management. Following this method, the review aims to contain as much existing knowledge as possible. 

### 3.3. Visualization of Similarities (VoS)

The VoS technique is used to map the interconnections and general trends in the collected data. This innovative approach facilitates a visual representation of complex relationships and trends, providing a view of current practices, research gaps, and emerging trends in the management of articular fractures of the foot.

### 3.4. Key Areas of Focus

The review places significant emphasis on several key areas:

- Innovative surgical techniques: Examining the latest advances in surgical procedures for treating articular fractures of the foot and their outcomes;
- Material science in orthopedics: Examining the role of new biocompatible materials and implants in fracture management;
- Rehabilitation and recovery: Investigating current trends in post-surgical rehabilitation and patient recovery, identifying the most effective practices;
- Technological innovations: Assessing the impact of technological innovations such as advanced imaging techniques, 3D imaging, robotic surgery, and biomaterials in bone healing.

### 3.5. Outcome and Ethical Considerations

This review aims to provide readers with an understanding of how foot fractures are currently managed, with a focus on effective treatments and identifying areas that require further exploration. This assessment ensures that all studies adhere to the highest ethical standards, protecting patient confidentiality and ensuring the accuracy of their data.

## 4. Results

### 4.1. What Are the Current Best Practices in the Management of Foot Fractures and Where Do They Fall Short?

**Figure 2:**
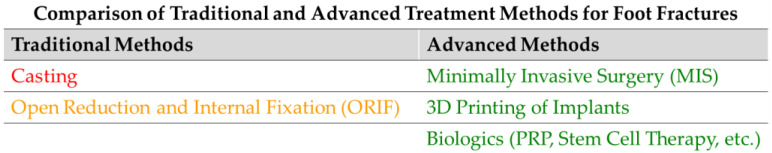
Type of treatment methods for foot fractures.

Articular fractures of the foot, especially those involving important joints such as the calcaneus and talus, represent significant challenges for orthopedic surgeons. To achieve the best possible outcomes for patients with these fractures, an understanding of surgical techniques and postoperative care is necessary. The main methods of their treatment are represented in Figure 2.

#### 4.1.1. Surgical Management: Open Reduction and Internal Fixation (ORIF)

**Figure 3:**
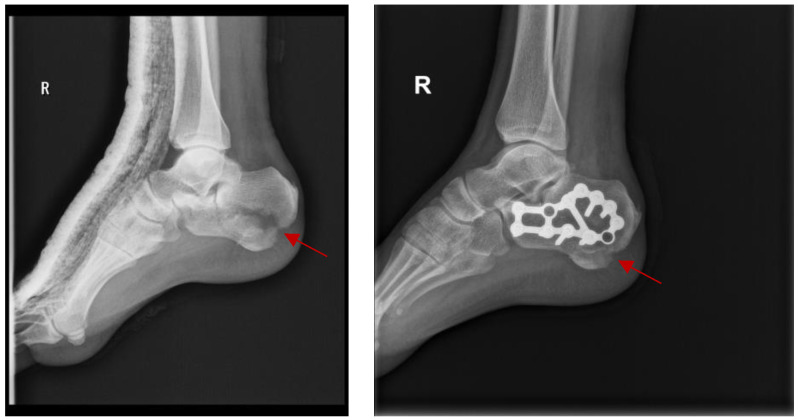
The fracture at the level of the calcaneus, initially treated with a cast that had not obtained the desired result and needed to be treated using ORIF method. The arrow shows the calcaneal fracture.

For physicians encountering displaced intra-articular fractures, the technique of internal reduction and fixation (ORIF) has gained widespread acceptance. Achieving correct anatomical alignment is a benefit of this method, as it is necessary to maintain the functionality of the joints and reduce the risk of post-traumatic arthrosis. In a study by Amr Selim et al. [9], functional outcomes of calcaneus fractures treated with open reduction and internal fixation (ORIF) were evaluated. An example of ORIF being necessary as a method of treatment for calcaneus fracture, where a cast was initially used for immobilization but did not fulfill the requirements for an optimal treatment, is presented in Figure 3.

The findings of the study highlight the importance of fixation to improve functionality and reduce the incidence of post-traumatic arthrosis, but it is specified that this must be taken into consideration, along with risk factors such as diabetes, smoking, peripheral vascular diseases, obesity, and alcoholism. Also, for the treatment of intra-articular fractures of the calcaneus, Steelman et al. [10] compared three methods: percutaneous reduction and fixation, open reduction, and internal fixation (ORIF), as well as combinations of two of these methods. Although both techniques produced satisfactory results, it was pointed out that ORIF was associated with a higher rate of infection, requiring another surgery. Therefore, it is necessary to carefully evaluate patient-specific factors before choosing this invasive procedure. In addition, a significant therapeutic challenge is the effective management of complications following intra-articular fractures of the distal tibia. The researchers Grazhdanov et al. [11] found that there must be a consistent treatment protocol that takes into account the many surgical options (such as ORIF), and their indications and contraindications. Finally, it is important to be cautious when using ORIF, and take into account patient characteristics and potential repercussions, even though it remains a basic strategy for treating displaced intra-articular fractures. The outcomes and challenges associated with this surgery are improved by the continuous evolution of surgical procedures and postoperative therapy tactics.

#### 4.1.2. Minimally Invasive Surgery (MIS)

**Figure 4:**
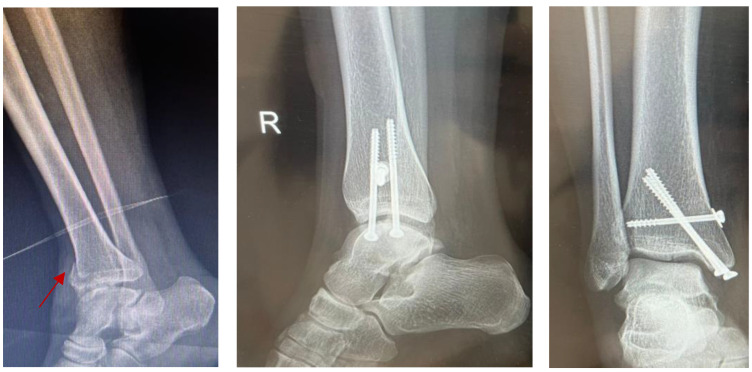
MIS used in treatment of tibial pillar fracture. The arrow shows the pillar fracture.

A less invasive technique that has grown in popularity recently is minimally invasive surgery (MIS). Kawade et al. [12] and Rayes et al. [13] showed that minimally invasive surgery, including percutaneous screw fixation, leads to better outcomes and fewer complications after surgery. The study by Zhao et al. [14] compared ORIF to the effectiveness of screw fixation and plate fixation using the tarsal sinus approach, as well as minimally invasive techniques, all of which are thoroughly evaluated in this study, suggesting that there are considerable advantages in some types of fractures, especially in terms of a minimal soft tissue damage and faster healing, as presented in Figure 4, where three percutaneous screws were used for the fixation of a tibial pillar fracture. 

Although MIS may help reduce soft tissue problems and accelerate healing, studies by Chotikkakamthorn et al. [15] and Lee et al. [16] show that the results may not always be as stable as with ORIF. This raises the question of how well it will work long-term, to repair complex fractures. With regard to making a decision between the benefits of less invasive surgery and the need for effective and durable fracture therapy, ever-evolving techniques continue to focus on improving minimally invasive surgery (MIS) in orthopedic surgery.

#### 4.1.3. External Fixation and Arthroscopic Technique

There have been recent advances in the management of calcaneal and peri-articular fractures regarding external fixation and arthroscopy techniques, representing new treatment methods where open reduction and internal fixation (ORIF) might be contraindicated. A notable contribution in this field is the study by Rodriguez-Collazo and Agyen [17], which introduces a novel approach using the Ilizarov method with the Orthofix Truelok circular external fixator for the reconstruction of Sanders type III and IV calcaneal fractures. This technique is very beneficial in cases where ORIF is contraindicated, such as in patients with compromised soft tissues or those at high risk of infection. Known for its versatility and adaptability, the Ilizarov method allows for precise fracture alignment and stabilization, promoting optimal healing while minimizing soft tissue damage. This study was conducted for 2.5 years, finding that only 8% of these surgeries had a poor result, meaning that this method may be a good alternative to ORIF. 

In the field of percutaneous interventions, the study by Patilet al. [18] compares the results of intra-articular calcaneal fractures treated with percutaneous cannulated cancellous screws versus ORIF performed with plating. This comparison is important because it highlights the potential of minimally invasive procedures in achieving comparable, if not superior, results compared to more traditional, invasive methods. The use of percutaneous screws provides a less invasive alternative, reducing the risk of postoperative complications such as wound infection and soft tissue damage, which are often concerns associated with ORIF. 

The study conducted by Kuloor et al. [19] on internal fixation of pillar fractures provides a comparative analysis of two treatment options, ORIF and minimally invasive plate osteosynthesis (MIPO). This study is particularly relevant because it highlights the evolution of fracture management, where less invasive techniques such as MIPO are increasingly used. MIPO, with minimal soft tissue dissection and reduced perioperative morbidity, presents a viable alternative to ORIF, especially in cases where preservation of soft tissue integrity is paramount. These recent studies highlight a shift toward less invasive, but highly effective surgical techniques in the management of calcaneal and peri-articular fractures. External fixation, particularly using advanced systems such as the Illizarov method, and minimally invasive procedures, such as percutaneous screw fixation and MIPO, are viable alternatives to ORIF. These advances not only provide improved outcomes in terms of reduced complications and improved functional recovery, but also expand the treatment options available to orthopedic surgeons, especially in complex cases where conventional methods may present significant risks.

#### 4.1.4. Non-surgical Management

**Figure 5:**
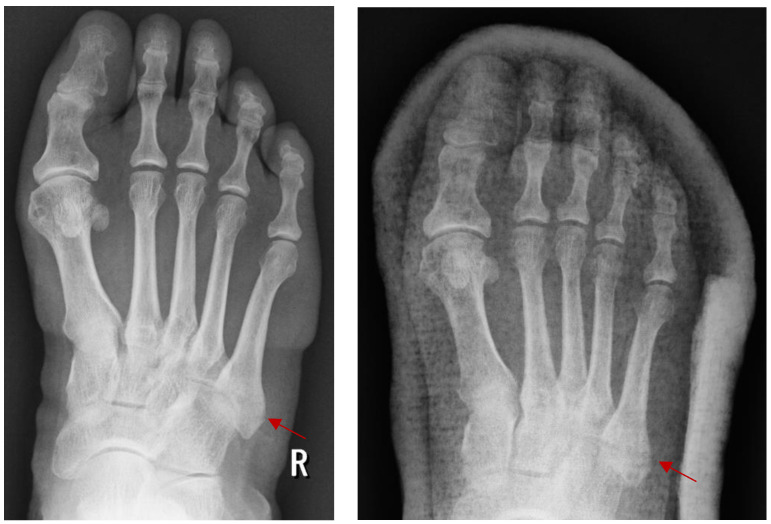
Fifth metatarsal fracture treated by immobilization in plaster. The arrow shows the fifth metatarsal fracture.

There are still differences between non-surgical and surgical approaches in terms of treating joint fractures, especially of the foot. For fractures that are not displaceable and are at high risk for surgery, the non-surgical approach, primarily involving physical therapy and immobilization, is essential. Patients with medical problems that may complicate surgery, or those who are at higher risk of postoperative complications, may benefit from this method, as shown in Figure 5, where a fifth metatarsal fracture without displacement is treated using plaster. 

The effectiveness of non-surgical treatments has been demonstrated by studies by Lee et al. [16] and Griffin et al. [20]. This research shows that, in certain situations, conservative treatment can have similar results to surgery in terms of pain management and functional recovery. Lee et al. [16] conducted an in-depth investigation of individuals who sustained non-displaced intra-articular calcaneal fractures. They found that patients who received conservative treatment for 12 months experienced improved mobility and a significant reduction in pain. Griffin et al. [20] also investigated a group of people with similar fractures. They found that the non-surgical approach was better in terms of pain management and daily activity, compared to those who had surgery. 

However, these studies show a significant problem with non-surgical management, which is the risk of developing arthrosis and joint instability in the long term. Research shows that while pain and immediate function can be effectively managed, the lack of surgery in certain types of fractures can lead to suboptimal joint alignment, resulting inchronic instability and an increase in degeneration of the joint. This shows how important it is to carefully select patients and make personalized treatment plans for them. 

Several important factors influence the decision-making process regarding surgical or non-surgical options. Assessing the type of fracture and its severity is essential. For example, conservative management may be used to treat fractures that have not affected any joints and are stable or non-displaced. Fractures that are displaced or cause significant damage to the joint usually require surgery to restore joint congruity and stability. The patient’s health is another essential aspect; because comorbidities can complicate surgical outcomes, non-surgical methods may be better for people with comorbidities such as diabetes, peripheral vascular disease, or osteoporosis. In addition, the decision may be influenced by the patient’s age, activity level, and overall health, as younger and more active individuals may require surgical stabilization to return to their previous activity levels. Recovery potential is also important. This includes not only the possibility of biological cure, but also the patient’s ability to adapt to rehabilitation protocols, their access to rehabilitation services, and their support system. In non-surgical management, effective physical therapy focuses on restoring range of motion, strengthening periarticular muscles, and improving proprioception to compensate for the lack of surgery. 

Consequently, the success rate of non-surgical treatment of articular fractures in the foot, involving physical therapy and immobilization, may vary from one patient to another. The type and severity of the fracture, the general health of the patient, and the rate of recovery are all included in these considerations. Thus, the biggest challenge is personalized treatment planning, which requires an in-depth understanding of each patient’s unique situation and requirements. Better guidelines and decision-making tools are expected as research in this area continues, to help clinicians optimize treatment strategies for their patients.

**Figure 6:**
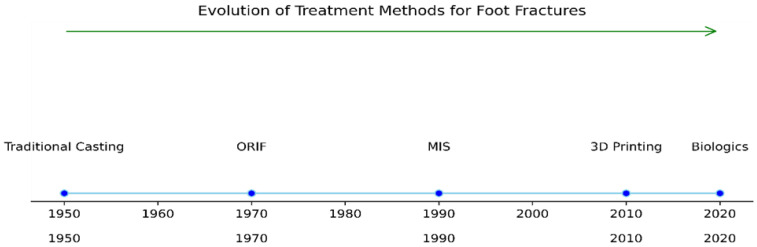
Evolution of the treatment of foot fractures, depending on the time they were used for the first time.

All of the methods are presented in the Figure 6, depending on when they were used for the first time.

#### 4.1.5. Rehabilitation in the Management of Articular Foot Fractures

The role of rehabilitation in the management of articular foot fractures is important. Individualized rehabilitation protocols are needed to improve functional recovery and reduce long-term disability, according to research by Obada et al. [21]. Taking into account type of fracture, surgery (if any), age, comorbidities and lifestyle, rehabilitation strategies must be tailored to the individual needs of each patient. An effective recovery process involves patient education, physiotherapy, and occupational therapy.

Dandale et al. [22] demonstrated a significant impact of physical therapy interventions on an efficient recovery. During the recovery phase, interventions that focus on restoring movement and strength help patients become more confident and independent. The method is very effective in preventing the long-term consequences of fractured bones.

Furthermore, the findings of Boileau et al. [23] demonstrate the importance of a personalized rehabilitation plan. Although they focus on shoulder fractures, their research highlights the relationship between patient characteristics and rehabilitation outcomes. This indicates that leg fracture rehabilitation requires an individualized approach, where all the particularities and requirements of each patient must be taken into account, to ensure the best possible recovery. 

Also, according to Hamedani et al. [24], technological advances reflect the evolution of rehabilitation. Highly effective rehabilitation strategies have resulted from their study of robot-assisted rehabilitation therapy, which are particularly useful in complicated cases of leg fractures.

Even though there are better ways to treat articular fractures in the foot, their treatment still remains a problem. Issues such as differences in access to healthcare, as discussed by Kawade et al. [12], and the need for more research regarding the long-term results of different treatment approaches, as pointed out by Rayes et al. [13], show how complex this field is. Also, the economic impact of these injuries, including the direct costs of medical care and the indirect costs related to loss of productivity and long-term disability, is still a major concern, as researched by Checa-Betegón et al. [25] and Falis and Pyszel [26]. This shows that more research needs to be performed regarding treatment methods, and more attention needs to be paid to public health, to reduce the effects of these injuries.

Additionally, the discussion of technological advances, such as robot-assisted therapy and computerized optical topography to monitor recovery, could be expanded to include wearables. Devices such as smart insoles and activity trackers are increasingly being used in the rehabilitation process, providing real-time feedback on weight distribution, walking patterns, and activity levels. This feedback enables better rehabilitation protocols and encourages patient involvement in the recovery process.

**Figure 7:**
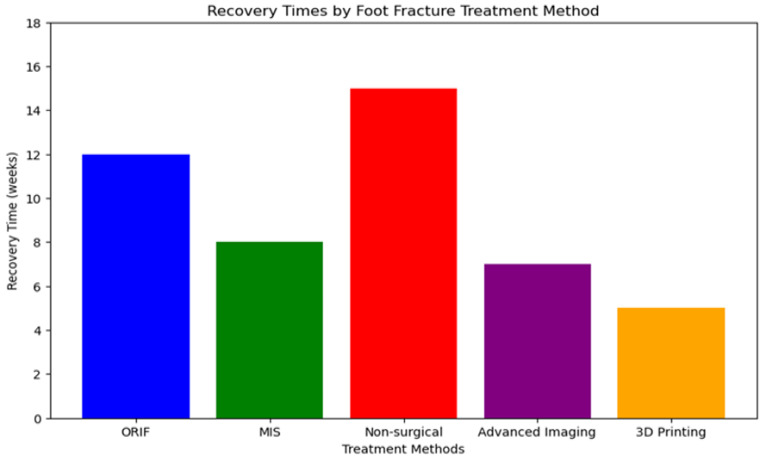
Recovery times depending on the method used for the treatment of foot fractures.

In conclusion, best practices for treating articular fractures in the foot have come a long way over the years, including both surgical and non-surgical methods, but they still have different levels of efficiency, as shown in Figure 7. However, they are not without limitations. Treatments must be individualized to each patient, based on multiple aspects (the type of fracture and functional needs). In order to fix current problems and improve the results for patients, a lot of research and development in this area is required, along with a focus on a patient-centered care.

#### 4.1.6. Challenges and Future Directions

The treatment of foot articular fractures can be difficult in multiple ways. The main problem of foot fractures is the fact that a high percentage of patients who suffer from such fractures are at an advanced age, as well as having a series of comorbidities. According to Clark D et al. [27], aging negatively affects the cellular and molecular processes (inflammatory regulation, cellular differentiation, and signaling cascades) that take place in the fracture healing process. Hasselman et al. [28] specified the fact that following the measurement of bone density, older patients with osteoporosis had more fractures in the foot than young, non-osteoporotic patients; however, patients with a high body mass index also had a higher percentage of ankle fractures. Thus, the advanced age of the patient, the possibility of having osteoporosis, and other factors that affect the healing processes of fractures, must be taken into account. In the treatment process, the mobility of the patient before the fracture must also be taken into account, as the elderly have reduced mobility due to joint arthrosis and muscle atrophy. The functional recovery of the affected segment after the end of the treatment can be long-term but, in some cases, patients are unable to regain the mobility they had before the fracture. Because of the complexity of fractures and the high potential for comorbidity, it is imperative to find a way to minimize soft tissue complications, while ensuring accurate surgical execution. The main problem of the existing approaches, even if they are effective, is that there is room for improvement through research and development. Because they are less invasive, non-surgical treatments can be used, but the results can be unsatisfactory, especially for displaced fractures. 

Future research should focus on identifying the best methods of treatment, achieving better outcomes for patients, and modifying the healthcare system to meet the ever-increasing demands. Ongoing research focuses on new surgical approaches, improved MIS solutions, and better postoperative recovery approaches.

Finally, the current methods of treatment of articular fractures, which include surgical and non-surgical methods, have made significant progress, but there are still many problems that can be improved. The type of fracture, general health, and functional needs of each patient determine the best treatment plan. We must continue to research and introduce new concepts in this field, put more emphasis on patient-centered care, and make health systems more efficient in order to prevent the problems that arise today and improve patient outcomes.

### 4.2. How Can Emerging Technologies and Innovations in Medical Science Be Integrated into the Treatment of Foot Fractures to Improve Clinical Outcomes?

The integration of emerging technologies and medical innovations is significantly enhancing the treatment of foot fractures, leading to improved clinical outcomes. Advanced imaging techniques, such as MRI and CT scans, have been pivotal in enhancing diagnostic accuracy, enabling tailored treatment plans. This advancement, as detailed by Soomekh [29], allows for a more precise assessment of the fracture and aids in formulating a more effective treatment strategy. Minimally invasive surgery (MIS), particularly the use of arthroscopy, minimizes tissue damage and accelerates the recovery process. This approach represents a significant shift towards less invasive surgical techniques in orthopedics.

The advent of 3D printing technology, as explored by Temple [30] and Plavitu et al. [31], is revolutionizing the creation of custom implants and surgical guides. This technology ensures a higher degree of precision in fracture management, adapting to the unique anatomical features of each patient. Concurrently, the field of biologics and tissue engineering, including the use of PRP and stem cell therapies, is emerging as a powerful tool for enhancing the body’s natural healing processes. These innovative therapies, as discussed by Temple [28], hold the potential to expedite recovery and improve the overall healing of foot fractures.

In the field of postoperative care, smart implants and wearable technologies are becoming increasingly important. These devices, capable of real-time monitoring of the healing process, optimize post-operative care and rehabilitation. Additionally, the integration of telemedicine and AI into postoperative care is reshaping the landscape of rehabilitation. These technologies offer personalized care plans and improved access to medical services.

Robotic-assisted surgery, another field advanced by Soomekh [29], offers unprecedented precision in surgical procedures, potentially leading to better alignment and stabilization of fractures. Complimenting this, virtual and augmented reality technologies are enhancing surgical training and planning, providing immersive environments for surgeons to hone their skills and plan complex procedures.

Nanotechnology, particularly in the development of orthopedic implants and drug delivery systems, is being investigated for its potential in improving implant integration and reducing infection rates. Temple’s [30] work sheds light on the promising applications of nanomaterials in orthopedics. In regenerative medicine, innovative approaches like bioactive scaffolds and growth factor delivery systems are showing promise in enhancing bone regeneration and repair.

Electromagnetic therapy, especially pulsed electromagnetic field therapy, is gaining attention for its potential role in enhancing fracture healing and treating conditions like tendinopathy, as explored by Gerdesmeyer [32]. Finally, the use of ortho-biologics, including bone graft substitutes and osteoconductive materials, is becoming more prevalent in the treatment of complex foot fractures, particularly in cases with bone loss.

These advancements collectively signify a paradigm shift towards more personalized, precise, and minimally invasive care in foot fracture management. They underscore the importance of continuous clinical research to validate these new technologies and to integrate them effectively into clinical practice.

### 4.3. What Role Do Healthcare Disparities Play in the Treatment of Foot Fractures, and How Can These Be Addressed to Ensure Equitable Care for All Patients?

Health care disparities influence foot fracture treatment and outcomes, showing a complex combination of socioeconomic, racial, and geographic factors. These disparities manifest in various aspects of health care, from access to treatment options to long-term outcomes. A study by Zelle et al. [33] of healthcare disparities among US orthopedic trauma patients showed that sociodemographic factors, such as age, gender, insurance status, race/ethnicity, and income, significantly impacted the use of surgical treatment for calcaneus fractures. This study, involving 17,156 patients, found that 41.03% received operative treatment, with decisions being influenced by these sociodemographic factors, as well as comorbidities such as diabetes, peripheral vascular disease, psychosis, drug abuse, and alcohol abuse. Hospital size and type also played an important role in the management of the fractures.

Driesman et al. [34], in their study of racial disparities in outcomes of operatively-treated lower extremity fractures, noted that minority patients, particularly African Americans and Hispanics, had worse long-term functional outcomes after surgery for lower extremity fractures. This was linked to factors such as high-velocity injury mechanisms and a higher incidence of open fractures among these groups, and their pain scores and functional outcome scores over 12 months were significantly worse. 

Rabah et al. [35] in their review, “*Are There Nationwide Socioeconomic and Demographic Disparities*”, highlighted differences in health care utilization, with lower ambulatory care and higher emergency department utilization among Hispanic patients, less educated patients, and those with lower incomes and no private insurance. This group’s healthcare expenditures for musculoskeletal conditions were substantially higher in the emergency department than in the orthopedic setting, highlighting the financial implications of these disparities.

Addressing these disparities requires several actions, including improving access to ambulatory care, especially for under-represented and disadvantaged populations, but this involves collaboration with policy-makers and initiatives for minority populations. Training health care providers to deliver care specifically for each cultural population can help them understand and address the unique needs of a diverse range of populations. Policy and health system reforms aimed at reducing financial barriers, such as expanding insurance coverage and reducing costs from patients’ own pockets, are essential to making medical care more accessible to all socioeconomic groups. Community awareness and education, particularly in disadvantaged areas, can help alleviate delays in seeking care. Continued research into the causes of these disparities, and effective strategies to address them, including the collection of patient outcome data based on various demographic factors, is also vital. 

Health disparities significantly affect the treatment and outcomes of foot fractures. Addressing them requires a comprehensive approach that includes improving access to care, improving cultural competency among health care providers, implementing policy reforms, community outreach, and continued research in this area. Such strategies are essential to ensure equal care for all patients, mitigating the impact of socioeconomic, racial, and geographic factors on treatments and outcomes.

### 4.4. How Do Post-Treatment Rehabilitation Strategies and Patient Adherence Affect the Long-Term Outcomes of Articular Foot Fracture Management?

Beyond surgery, long-term follow-up in the management of foot articular fractures depends on post-treatment rehabilitation techniques and patient compliance. This model places a strong emphasis on the influence of socioeconomic conditions, early mobilization, personalized rehabilitation, and psychological support.

Early mobilization and rehabilitation: Lin et al. [36] state the significance of early mobilization in patients with foot fractures. Their findings indicate that early weight-bearing exercises, as tolerated, not only enhance functional outcomes, but also decrease the recovery time;

Customized rehabilitation programs: According to Chiodo et al. [37], rehabilitation programs ought to be customized based on the individual requirements of patients and the kind of fracture they have sustained. Such personalized programs improve patient satisfaction and functional rehabilitation. This customized strategy guarantees that rehabilitation is a patient-centered strategy that considers the various demands and situations of each patient;

Patient adherence to rehabilitation protocols: Smith et al. [38] address the significance of patient adherence to recommended rehabilitation protocols. According to their research, adherence is associated with favorable results like improved pain relief, increased range of motion, and improved foot function overall. This demonstrates the significance of patient involvement and education in the rehabilitation process, as it guarantees patients’ motivation and helps them understand the advantages of following their treatment plan;

Psychological aspects of rehabilitation: Moseley et al. [39] studied the psychological dimensions of rehabilitation, finding that a positive mindset and active participation significantly contribute to better functional outcomes and a quicker return to daily activities. This aspect of rehabilitation acknowledges the mind-body connection and the role of mental health in physical recovery; 

Impact of socioeconomic factors: Hunt et al. [40] reveal that socioeconomic factors influence patient adherence and rehabilitation outcomes. Patients with better access to healthcare resources and support systems are more likely to experience favorable long-term outcomes following foot fracture treatment. This highlights the need for equitable healthcare access and the importance of addressing socioeconomic disparities in healthcare; 

Influence of surgical approach: Research by Marin et al. [41] and Schepers et al. [42] compared the outcomes of two surgical techniques for calcaneal fractures, the extended lateral approach and the less invasive sinus tarsi approach. Their findings suggest that less invasive techniques can achieve similar functional outcomes to traditional approaches, which could influence postoperative rehabilitation strategies;

Customized treatment approaches: Zhang et al. [43], in a study not directly related to foot fractures but relevant in its emphasis on customized treatment, highlighted the importance of individualized approaches in medical practice. This principle is applicable to foot fracture management, where personalized rehabilitation strategies are essential for optimal outcomes Moga et al. [44,45]. 

Thus, these studies collectively suggest that successful post-treatment rehabilitation oof foot fractures is strongly influenced by early and active mobilization, customized rehabilitation programs, patient adherence, psychological well-being, socioeconomic factors, and the initial surgical approach. They emphasize the need for a patient-centered approach, both in the surgical phase and in the rehabilitation phase, to achieve better long-term outcomes. This perspective is important in the evolving field of orthopedic care, where the goal is not only to repair the fracture, but also to restore the patient’s quality of life and functionality to the greatest extent possible.

### 4.5. Hypotheses and Findings: A Synergistic Evaluation

Alignment of hypotheses with findings.

1. Technological advancements and healthcare disparities:

The research results provide substantial support for the first hypothesis. Recent advances in medical technology, particularly minimally invasive surgery (MIS), 3D printing, and biologics, have been observed to significantly improve treatment outcomes. However, a disparity has been observed in their application. The results indicated a marked disparity in access to these technologies, often influenced by geographic and socioeconomic factors, thus validating the hypothesis that while technological advances are revolutionizing treatment, their full potential is hindered by existing health disparities;

2. Limitations of current treatment methodologies:

The second hypothesis assumes that current treatment methodologies, although fundamental, have inherent limitations in addressing the nature of foot fractures. The research reveals that traditional approaches such as open reduction and internal fixation (ORIF), although effective, do not universally respond to the complexity of different fracture types. The findings highlight the need for innovative approaches that are adaptable to individual patient profiles, taking into account factors such as biomechanics, patient health, and post-treatment rehabilitation. This observation confirms the hypothesis, emphasizing the need for personalized, patient-centered treatment plans. 

The concordance of these hypotheses with research findings highlights a vital aspect of modern orthopedic practice, the need for continued innovation and personalized care in the treatment of foot fractures. While technological advances offer promising avenues for improved treatment outcomes, their effectiveness depends on equitable access and integration into clinical practice. Similarly, the reformulation of existing treatment methodologies to include patient-specific considerations is imperative to address the diverse challenges presented by different types of foot fractures. This research not only validates the initial hypotheses, but also provides a roadmap for future advances in the field, highlighting the need for a more nuanced and patient-focused approach to the management of foot fractures.

## 5. Future Perspectives

### 5.1. Emerging Technologies and Personalized Medicine

Advances in 3D printing, biologics, and minimally invasive surgical techniques are set to increase precision in fracture management, offering customized solutions that take into account the anatomy of each individual patient, as well as the specific characteristics of each fracture. The potential benefits of integrating artificial intelligence into diagnostic and therapeutic decision-making processes cannot be overstated. These technologies, along with advances in implant and prosthetic materials science, are expected to significantly improve functional outcomes and accelerate recovery times.

### 5.2. Addressing Healthcare Disparities

An important focus for future research and policy development should be addressing the health disparities that currently limit access to these advanced treatments. Efforts must be directed towards ensuring that the entire population can access these revolutionary treatment technologies. This involves not only overcoming socioeconomic and geographic barriers, but also integrating cultural competencies into patient care, thus ensuring that the benefits of medical advances are universally available.

### 5.3. Biomechanics and Rehabilitation

The evolving understanding of foot biomechanics will continue to refine surgical techniques, leading to better alignment, stability, and long-term joint functionality. At the same time, the role of rehabilitation in the management of foot fractures will gain even more importance. Personalized rehabilitation protocols, that take into account the specific type of fracture, surgery, and individual patient factors, will be essential to ensure optimal recovery. The incorporation of telerehabilitation and wearable technology for continuous monitoring and adaptation of rehabilitation protocols is likely to become more widespread.

### 5.4. Interdisciplinary Collaboration and Patient-Centered Care

Future directions in the management of foot fractures will also emphasize the importance of interdisciplinary collaboration. Integrating knowledge from orthopedics, biomechanics, materials science, and rehabilitation specialists will be essential when developing treatment strategies. Patient-centered care, involving patients in decision-making processes, and tailoring treatments to meet their specific needs and lifestyle considerations, will also be essential. This approach not only respects patient autonomy, but also ensures better adherence to treatment and rehabilitation protocols, leading to improved outcomes.

### 5.5. Research and Clinical Trials

**Figure 8:**
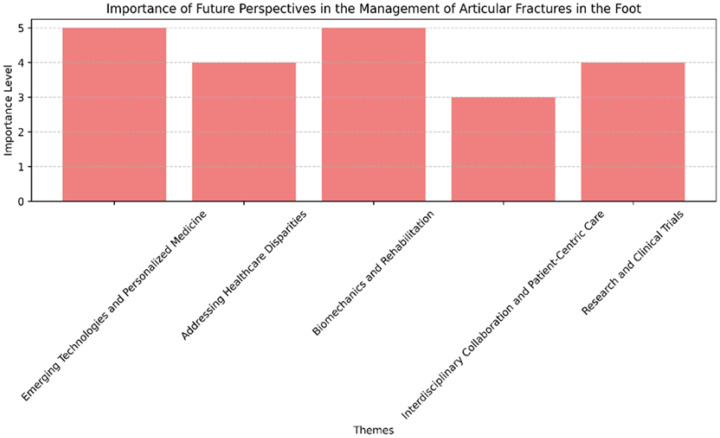
Future perspectives depending on their importance.

Continued research and well-designed clinical trials will be essential in validating new treatments and technologies. These studies should focus not only on the efficacy and safety of new approaches, but also on long-term outcomes, including quality of life and functional recovery. Comparative studies between traditional and new treatment modalities will provide valuable information, guiding clinicians in choosing the most effective treatment strategy. Figure 8 shows a representation of which fields scientists should focus on, depending on their importance. 

## 6. Conclusions

This research, focused on the management of articular fractures in the foot, has highlighted significant problems to inform future advances in orthopedic care. The study began with hypotheses focused on the transformative potential of emerging medical technologies and the need for innovation in current treatment methodologies, given the diverse and complex nature of foot fractures. These assumptions were then substantiated by findings from a literature review.

Key Findings and Implications:

1. Technological advancements: Research highlights the critical role of technological advances, such as minimally invasive surgery (MIS), 3D printing, and biologics, in improving treatment outcomes. However, it also highlights disparities in access to these technologies, highlighting the need for equitable healthcare systems that can integrate these advances effectively, for all patients;
2. Treatment methodologies: The study confirms that while traditional treatment methodologies form the basis of foot fracture management, they are often limited in addressing the complexities of different fracture types. This validates the need for innovative, patient-centered treatment approaches that are adaptable to individual patient profiles, taking into account biomechanical perspectives and post-treatment rehabilitation;
3. Interdisciplinary collaboration: The findings argue for an interdisciplinary approach to the management of foot fractures, integrating perspectives from orthopedics, biomechanics, materials science, and rehabilitation, to develop comprehensive, patient-centered treatment strategies.

The future of foot fracture management lies in the successful combination of technological innovation, personalized medicine, and patient-centered care. Continued research and well-designed clinical trials are imperative to validate new treatments and technologies, focusing on efficacy, safety, and long-term outcomes. The study highlights the critical role of addressing health disparities, to ensure that the benefits of medical advances are universally accessible. The evolution of treatment modalities, based on biomechanical perspectives and patient-specific factors, is essential to improve the long-term outcomes and quality of life of patients with leg fractures. In conclusion, this research provides a comprehensive assessment of current methodologies in the management of foot fractures and outlines a visionary path for future advances. It emphasizes the need for continuous innovation, interdisciplinary collaboration, and a holistic approach to patient care. By addressing the identified challenges and harnessing the potential of emerging technologies, the field of orthopedic care can significantly improve the management of foot fractures, ultimately transforming patient outcomes and raising the standard of care in this complex and evolving field.

## References

[1] Aru R.G., Tyagi S. (2022). Endovascular treatment of femoropopliteal arterial occlusive disease: Current techniques and limitations. Semin. Vasc. Surg..

[2] Kim J., Radkievich R., Mizher R., Shaffrey I., O’malley M., Deland J., Demetracopoulos C., Ellis S. (2023). Outcomes of Total Ankle Arthroplasty in Postfracture Ankle Arthritis. Foot Ankle Int..

[3] Spira B. (2022). The Drive for Health Equity—The Need to Use Technology to Reduce Healthcare Disparities in Orthopedics. J. Orthop. Exp. Innov..

[4] Hofmann U.K., Hildebrand F., Mederake M., Migliorini F. (2023). Telemedicine in orthopaedics and trauma surgery during the first year of COVID pandemic: A systematic review. BMC Musculoskelet. Disord..

[5] Giorgino R., Alessandri-Bonetti M., Luca A., Migliorini F., Rossi N., Peretti G.M., Mangiavini L. (2023). ChatGPT in orthopedics: A narrative review exploring the potential of artificial intelligence in orthopedic practice. Front. Surg..

[6] Bolovan A.D., Onofrei R.R., Hogea G.B., Abu-Awwad A., Lazarescu E.A., Abu-Awwad S.A., Tapardea A.R., Suba M.I., Amaricai E.C. (2023). Comparison between Exercise Program-Foot Orthoses Treatment and Exercise Program Alone after Pilon Fracture Surgery: Study Protocol for a Randomized Controlled Trial. Life.

[7] Gouzoulis M.J., Joo P.Y., Kammien A.J., McLaughlin W.M., Yoo B., Grauer J.N. (2022). Risk factors for venous thromboembolism following fractures isolated to the foot and ankle fracture. PLoS ONE.

[8] Rounds A.D., Burtt K.E., Leland H.A., Alluri R.K., Badash I., Patel K.M., Carey J.N. (2019). Functional outcomes of traumatic lower extremity reconstruction. J. Clin. Orthop. Trauma..

[9] Selim A., Ponugoti N., Chandrashekar S. (2022). Systematic Review of Operative vs Nonoperative Treatment of Displaced Intraarticular Calcaneal Fractures. Foot Ankle Orthop..

[10] Steelman K., Bolz N., Feria-Arias E., Meehan R.E. (2021). Evaluation of patient outcomes after operative treatment of intra-articular calcaneus fractures. SICOT-J.

[11] Grazhdanov K.A., Zuev P.P., Kauts O.A., Romanov N.I., Barabash Y.A., Kireev S.I., Norkin I.A. (2021). Surgical rehabilitation of patients with the consequences of pilon fractures. N.N. Priorov J. Traumatol. Orthop..

[12] Kawade M.S., Madan H.S., Shailesh Khachane M.S.D. (2019). Calcaneal fractures: A management dilemma-minimally invasive approach for intra and extra articular calcaneal fractures. Int. J. Res. Med. Sci..

[13] Rayes J., Sharplin P., Maalouf P., Willms S., Dodd A. (2023). A Stepwise Minimally Invasive Sinus Tarsi Approach to Open Reduction and Internal Fixation of Displaced Intra-articular Calcaneal Fractures: Technique Tip. Foot Ankle Int..

[14] Zhao B., Xu X., Sun Q., Liu Y., Zhao Y., Wang D., Gao Y., Zhou J. (2024). Comparison between screw fixation and plate fixation via sinus tarsi approach for displaced intra-articular calcaneal fractures: A systematic review and meta-analysis. Arch. Orthop. Trauma. Surg..

[15] Chotikkakamthorn N., Chanajit A., Tharmviboonsri T., Chuckpaiwong B., Harnroongroj T. (2021). Minimal invasive surgery in the management of intra-articular calcaneal fractures: A retrospective comparison of screw fixation alone versus screw with small locking plate fixation techniques. Acta Orthop. Traumatol. Turc..

[16] Lee H.S., Lewis D.P., Balogh Z.J. (2024). Supplementary medial plating in revision surgery for distal femoral fractures: A surgical technique with clinical outcomes. Injury.

[17] Rodriguez-Collazo E., Agyen J. (2018). Peri-Articular Reconstruction for Intra-Articular Calcaneal Fractures Utilizing the Ilizarov Method with Orthofix Truelok Circular External Fixator: A Technique Guide and Orthoplastic Considerations. https://www.semanticscholar.org/paper/Peri-Articular-Reconstruction-for-Intra-Articular-A-Rodriguez-Collazo-Agyen/59b16339acdb31e889378a770d6665195b3de72d.

[18] Patil P.V., A Nikam M., Shirahatti S., Kumar P. (2021). Effect of lateral wedged insole shoe in knee osteoarthritis: A 12 month randomized controlled trial. Int. J. Orthop..

[19] Kuloor S.B., Shareef A.J. (2019). Internal fixation of low energy pilon fractures: Prospective study of two treatment options (ORIF/MIPO). Int. J. Orthop. Sci..

[20] Griffin D., Parsons N., Shaw E., Kulikov Y., Hutchinson C., Thorogood M., Lamb S.E., UK Heel Fracture Trial Investigators (2014). Operative versus non-operative treatment for closed, displaced, intra-articular fractures of the calcaneus: Randomised controlled trial. BMJ.

[21] Obada B., Georgeanu V.-A., Popescu I.-A., Iliescu M.-G., Stanciu L.-E., Caraban B.M.C. (2023). Late functional and radiological outcomes in recovery of patients with staged osteosynthesis for the tibial pilon fractures. Balneo PRM Res. J..

[22] Dandale C., Chitale N.V., Phansopkar P. (2023). Effect of Physiotherapeutic Rehabilitation on a Patient With an Iliac Fracture, and Superior and Inferior Pubic Rami Fracture With Foot Drop: A Case Report. Cureus.

[23] Boileau P., Caligaris-Cordero B., Payeur F., Tinsi L., Argenson C. (1999). Facteurs pronostiques au cours de la rééducation après prothèses d’épaule pour fracture [Prognostic factors during rehabilitation after shoulder prostheses for fracture]. Rev. Chir. Orthop. Reparatrice Appar. Mot..

[24] Hamedani M., Prada V., Tognetti P., Leoni V., Schenone A. (2022). Robot-assisted and traditional intensive rehabilitation therapy in the treatment of post-acute stroke patient: The experience of a standard rehabilitation ward. Neurol. Sci..

[25] Checa-Betegón P., Valle-Cruz J.A., Llanos-Sanz S., Miguel-Miguel C., Sánchez-del-Saz J., García-Coiradas J. (2023). External fixation in intra-articular fractures of the calcaneus: Is it a valid option as definitive treatment?. Eur. J. Orthop. Surg. Traumatol..

[26] Falis M., Pyszel K. (2016). Treatment of Displaced Intra-articular Calcaneal Fractures by Intramedullary Nail. Preliminary Report. Ortop. Traumatol. Rehabil..

[27] Meinberg E.G., Clark D., Miclau K.R., Marcucio R., Miclau T. (2019). Fracture repair in the elderly: Clinical and experimental considerations. Injury.

[28] Hasselman C.T., Vogt M.T., Stone K.L., Cauley J.A., Conti S.F. (2003). Foot and Ankle Fractures in Elderly White Women: Incidence and Risk Factors. J. Bone Jt. Surg..

[29] Soomekh D.J. (2011). New technology and techniques in the treatment of foot and ankle injuries. Clin. Podiatr. Med. Surg..

[30] Temple H.T., Malinin T.I. (2016). Orthobiologics in the Foot and Ankle. Foot Ankle Clin..

[31] Plavitu A., Pogarasteanu M.E., Moga M., Lupusoru M., Radu F.I., Edu A. (2018). 3D Printing as a Way of Integrating Mathematical Models in Arthroscopic Knee Surgery. Rev. Chim..

[32] Gerdesmeyer L., Saxena A., Klueter T., Harrasser N., Fullem B., Krath A. (2017). Electromagnetic Transduction Therapy for Achilles Tendinopathy: A Preliminary Report on a New Technology. J. Foot Ankle Surg..

[33] Zelle B.A., Morton-Gonzaba N.A., Adcock C.F., Lacci J.V., Dang K.H., Seifi A. (2019). Healthcare disparities among orthopedic trauma patients in the USA: Socio-demographic factors influence the management of calcaneus fractures. J. Orthop. Surg. Res..

[34] Driesman A., Fisher N., Konda S.R., Pean C.A., Leucht P., Egol K.A. (2017). Racial disparities in outcomes of operatively treated lower extremity fractures. Arch. Orthop. Trauma. Surg..

[35] Rabah N.M., Knusel K.D., Khan H.A., Marcus R.E. (2020). Are There Nationwide Socioeconomic and Demographic Disparities in the Use of Outpatient Orthopaedic Services?. Clin. Orthop. Relat. Res..

[36] Lin J., Xie C., Chen K., Sun S., Zhou K., Zhou C., Kong J. (2019). Comparison of sinus tarsi approach versus extensile lateral approach for displaced intra-articular calcaneal fractures Sanders type IV. Int. Orthop..

[37] Chiodo C.P., Glazebrook M., Bluman E.M., Cohen B.E., Femino J.E., Giza E., Watters W.C., Goldberg M.J., Keith M., Haralson R.H. (2010). Diagnosis and treatment of acute Achilles tendon rupture. JAAOS J. Am. Acad. Orthop. Surg..

[38] Smith M., Medlock G., Johnstone A.J. (2017). Percutaneous screw fixation of unstable ankle fractures in patients with poor soft tissues and significant co-morbidities. Foot Ankle Surg..

[39] Moseley G. (2005). Lorimer. Is successful rehabilitation of complex regional pain syndrome due to sustained attention to the affected limb? A randomised clinical trial. Pain..

[40] Hunt K.J., Anderson R.B. (2011). Treatment of Jones fracture nonunions and refractures in the elite athlete: Outcomes of intramedullary screw fixation with bone grafting. Am. J. Sports Med..

[41] Marin M.I., Robert S., Sakizlian R.E., Rusu L., Rusu R.M. (2024). A Biomechanical Evaluation of the Upper Limb Kinematic Parameters of the Throwing Action in Handball: A Case Study. Appl. Sci..

[42] Schepers T. (2019). Sinus tarsi approach with screws-only fixation for displaced intra-articular calcaneal fractures. Clin. Podiatr. Med. Surg..

[43] Zhang X., Wang H., Du C., Fan X., Cui L., Chen H., Deng F., Tong Q., He M., Yang M. (2022). Custom-molded offloading footwear effectively prevents recurrence and amputation, and lowers mortality rates in high-risk diabetic foot patients: A multicenter, prospective observational study. Diabetes Metab. Syndr. Obes. Targets Ther..

[44] Moga M., Pogarasteanu M.E., Edu A. (2018). Arthroscopy in Arthrosis: Is It Worth it? A case Presentation. Rev. Chim..

[45] Moga M., Pogarasteanu M.E., Edu A. (2018). Arthroscopic Equipment Used in the Treatment of Calcaneal Spurs A case presentation. Rev. Chim..

